# Me_2_SO perfusion time for whole-organ cryopreservation can be shortened: Results of micro-computed tomography monitoring during Me_2_SO perfusion of rat hearts

**DOI:** 10.1371/journal.pone.0238519

**Published:** 2020-09-02

**Authors:** Nathalie Bleisinger, Ralf Dittrich, Olga Strahl, Robert Brauweiler, Inge Hoffmann, Matthias W. Beckmann, Tilmann Volk

**Affiliations:** 1 Department of Obstetrics and Gynaecology, Erlangen University Hospital, Friedrich-Alexander University Erlangen-Nürnberg, Erlangen, Germany; 2 Institute of Medical Physics, Friedrich-Alexander University Erlangen-Nürnberg, Erlangen, Germany; 3 Institute of Cellular and Molecular Physiology, Friedrich-Alexander University Erlangen-Nürnberg, Erlangen, Germany; Faculty of Animal Sciences and Food Engineering, University of São Paulo, BRAZIL

## Abstract

Cryopreservation of whole organs and specific tissues is an important and continually expanding field of medicine. The protocols currently used for organ preservation do not ensure survivability and functionality; the protocols for ovarian tissue lead to acceptable outcomes, but these are still capable of further improvement. In general, cryopreservation protocols need to be optimized. One important approach to improving cryopreservation protocols in general involves reducing exposure to cytotoxic cryoprotective agents prior to freezing. This study, therefore, evaluated the real-time tissue penetration of dimethyl sulfoxide, a cryoprotective agent that is widely used in cryopreservation. Dimethyl sulfoxide penetration in rat hearts perfused with a 15% (v/v) dimethyl sulfoxide solution was examined in real-time using dynamic contrast-enhanced micro-computed tomography imaging. Viability of cardiomyocytes was not significantly affected by the dimethyl sulfoxide perfusion procedure. Two different perfusion rates were evaluated and compared with perfusion using a common iodine-based contrast agent (iomeprol). The dynamic contrast-enhanced micro-computed tomography imaging data showed that dimethyl sulfoxide flushes both the extracellular and intracellular spaces in rat heart tissue to 95% equilibration after ≈ 35 s via perfusion. Subsequent wash-out via perfusion is completed to 95% within ≈ 49 s. The equilibration duration routinely used in dimethyl sulfoxide–based protocols for cryopreservation should therefore be questioned. Shorter incubation duration would perhaps be sufficient, as well as being beneficial in relation to cell survivability. It would be helpful to have techniques for non-invasive real-time monitoring of the penetration of cryoprotective agents and such techniques should be used to revise cryopreservation protocols. Switching to perfusion-based equilibration procedures might be beneficial, if feasible.

## Introduction

Since the discovery of cryoprotective agents (CPAs) in 1949 [1], they have been used in a range of cryopreservation applications to protect single cells, tissue and/or whole organs from damage arising during freezing to very low temperatures and subsequent warming procedures. CPAs are mainly used to prevent the extracellular and intracellular formation of ice crystals. Intracellular ice formation, in particular, is considered to be detrimental as intracellular ice crystals could pierce the cell membrane as well as intracellular structures [2].

Dimethyl sulfoxide (Me_2_SO) was introduced as a cryoprotectant during cryopreservation procedures by Lovelock and Bishop in 1959 [3]. Since then, Me_2_SO has been tested and routinely used for a wide range of applications in cryobiology (i.e., freezing of cell suspensions, gametes, embryos, tissues and whole organs). However, exposure to Me_2_SO is in general cytotoxic, and the same also applies to other CPAs [4]. The toxicity of Me_2_SO increases with elevated concentrations, temperatures and exposure duration; consequently, the concentration of CPA that can be used and the exposure duration during cryopreservation are limited. The necessary challenge is to find the right balance between achieving sufficient cryoprotection through dehydration and an acceptable degree of toxic damage, and this has still not yet been optimized [4]. Another important aspect is to ensure that all parts of the sample are penetrated by the CPA [2], and this is difficult to achieve in larger tissue pieces and whole organs.

We hypothesized that the time it takes for Me_2_SO to be distributed through tissue may be shorter than expected. Therefore this study evaluated the penetration time of Me_2_SO during perfusion of whole rat hearts, using imaging with simultaneous dynamic contrast-enhanced (DCE) micro-computed tomography (micro-CT). DCE micro-CT proved to be an excellent method for examining this hypothesis, as it allows simultaneous real-time observation of the distribution of Me_2_SO inside the organ. We show that by arterial perfusion Me_2_SO quickly and completely distributes in the tissue within 30-40s and can be removed by perfusion almost equally quickly. The information obtained about the entry of Me_2_SO into cells during perfusion may be important for improving tissue and organ cryopreservation protocols.

## Materials and methods

### Animals

Thirteen adult female Wistar rats (Charles River Laboratories Inc., Wilmington, Massachusetts, USA), aged 8–12 weeks, 200–250 g body weight, were housed with water and standard pellet food ad libitum. The housing conditions were standardized: temperature 20 ± 2°C, humidity 50 ± 5% and a 12-h light/dark rhythm. The study was conducted after approval from the ethical committee on animal experimentation in 2005 (District Council of Mittelfranken, Ansbach, Germany, reference number 621–2531.32-11/05). Animals were maintained in accordance with Animal Care and Use Committee regulations.

### Organ harvesting

Removal of the heart and initial perfusion followed a procedure published previously [5]. Briefly, animals were sacrificed by removal of the heart in deep anesthesia, induced by intraperitoneal injection of thiopental sodium (100 mg/kg body weight). The heart was quickly excised via a median sternotomy and placed in cold (4°C) modified Tyrode’s solution, in which it stopped beating immediately. The modified Tyrode’s solution contained: 138 mM NaCl, 4 mM KCl, 1 mM MgCl_2_, 0.33 mM NaH_2_PO_4_, 10 mM glucose and 10 mM *N*-2-hydroxyethylpiperazine-*N*-2*-*ethanesulfonic acid (HEPES) buffer. It was modified by adding 5 mM ethyleneglycoltetraacetic acid (EGTA) and 4.5 mM CaCl_2_, adjusted to a pH of 7.30 with NaOH. The heart weight (including residual parts of large vessels) ranged from 1.0–1.4 g.

### Perfusion (wash-in), DCE micro-CT and imaging protocol

Immediately after harvesting, the aortic root was cannulated with a 14-gauge intravenous cannula. The heart was briefly perfused using modified Tyrode’s solution containing 5 mM EGTA and 4.5 mM Ca^2+^ (free Ca^2+^ concentration ≈ 1 μM) to remove blood from the vessels and Ca^2+^ from the extracellular space, to prevent contraction. The heart was stored on ice for up to 3 h until further experiments. Perfusion with the radiographic contrast medium iomeprol (Imeron 400; Bracco Imaging Deutschland GmbH, Constance, Germany) or with dimethyl sulfoxide (Me_2_SO) was carried out using a microinjector (KDS 200P, ISMATEC GmbH, Wertheim, Germany) that allowed a constant flow at a rate of 5 or 10 mL/min. The perfusion rates for Me_2_SO were 5 mL/min and 10 mL/min, respectively; the iomeprol solution was perfused at 5 mL/min. The perfusion temperature was 22 ± 1°C; the maximum arterial perfusion pressures were 64–110 mmHg during 5 mL/min perfusion and 138–268 mmHg at 10 mL/min.

The DCE micro-CT protocol was similar to a procedure published previously [6]. Briefly, the hearts were placed in the center of a DCE micro-CT field of view for simultaneous visualization of the organ perfusion. DCE micro-CT was used to evaluate contrast enhancement during Me_2_SO or iomeprol perfusion, as well as the decrease in contrast during the wash-out process; CT values are given in Hounsfield units (HU). HUs are units on the Hounsfield scale, normalizing attenuation levels with those of water (0 HU) and air (–1000 HU). All of the images were acquired using a dual-source desktop micro-CT scanner (TomoScope Duo, CT Imaging GmbH, Erlangen, Germany) in standard quality mode at a tube current of 300 μA, a tube voltage of 65 kV, and with a 40*-*mm field of view. The system uses slip-ring technology to support dynamic CT. The perfusion acquisition consisted of 30 full continuous rotations of the gantry; the rotation time was 4 s, resulting in a total acquisition time of 120 s. Overlapping reconstructions were performed at 2*-*s intervals with full imaging data over a 360° range, resulting in 58 image sets for all wash-in and wash-out procedures. In all, 100 total projection view angles were used in each image reconstruction. The reconstructions were set in such a way as to obtain axial images on a 512 × 512 grid with an isotropic voxel size of 81 μm. The first reconstructed image was used as a baseline image.

### Me_2_SO and iomeprol dilutions

Me_2_SO was diluted with modified Tyrode’s solution, supplemented with 5 mM EGTA and 4.5 mM Ca^2+^ (free Ca^2+^ concentration ≈ 1 μM) to 15% (v/v) Me_2_SO. The final 15% (v/v) Me_2_SO solutions reached about 450 HU on CT imaging (solution only).

Iomeprol is a nonionic monomeric molecule that is used as a contrast medium during CT imaging. Iomeprol distributes intravascularly as well as in the extracellular space and does not enter the cells [7]. Therefore, Iomeprol served as a reference substance for evaluating whether Me_2_SO distributes in different ways during perfusion, e.g. by entering intracellular spaces. For this purpose, the two substances were adjusted to the same CT value (solutions measured before perfusion). A dilution of 1:40 of iomeprol 400 (iomeprol concentration 20.41 mg/ml, corresponding to an iodine concentration of 10 mg/ml) was necessary to reach approximately 450 HU in the CT value (solution only), which is similar to the HU of 15% (v/v) Me_2_SO (solution only). The CT value was checked for each solution before perfusion. Iomeprol 400 was diluted with the same diluent as Me_2_SO (modified Tyrode’s solution, supplemented with 5 mM EGTA and 4.5 mM Ca^2+^).

### Wash-out

The Me_2_SO and iomeprol were washed out via perfusion with Me_2_SO/iomeprol diluent only (modified Tyrode’s solution, supplemented with 5 mM EGTA and 4.5 mM Ca^2+^) immediately after perfusion with Me_2_SO or iomeprol. The perfusion rate during wash-out was the same as during the preceding wash-in.

### Cell viability analysis

To exclude the possibility that either Me_2_SO -perfusion during the wash-in or Me_2_SO -removal during the wash-out led to substantial damage to cardiomyocytes, we performed control experiments in which hearts were perfused with identical solutions in the exact order while the perfusate was collected and assessed for the cardiomyocytes-specific isoform of creatine kinase (CK). CK-levels in the perfusate were determined using a Reflotron CK assay (Roche). After removal and initial perfusion with the modified Tyrode’s solution, no CK (detection threshold of the assay: 10 U/L) was detectable in the perfusate. This was also the case in the Me_2_SO perfusate (10 mL in 2 min). In the perfusate during the wash-out of Me_2_SO (10 mL in 2 min), CK was detectable in two out of three experiments, but at a very low level of 22 and 17 U/L. To verify that the CK-assay could detect CK at all (and also in the presence of 15% Me_2_SO) we destroyed the cardiomyocytes in the heart by a freeze-thaw cycle and perfused again with 10 mL of modified Tyrode’s solution within 2 min. In this perfusate, CK-levels were above the upper threshold of the Reflotron CK-assay. Therefore, this solution was diluted 1:100 or 1:10 with modified Tyrode’s solution or modified Tyrode’s solution containing 15% Me_2_SO before CK-levels were determined. CK-levels were not affected by the presence of 15% Me_2_SO, were calculated depending on the dilution factor and averaged 3320 +/- 756 U/L (n = 3). This demonstrates that the cardiomyocytes were not significantly affected by Me_2_SO -wash-in and wash-out under our experimental conditions.

### Experimental design and statistical analysis

Thirteen animals served as heart donors; four hearts were perfused with 5 mL/min Me_2_SO, three hearts were perfused with 5 mL/min iomeprol, three hearts were perfused with 10 mL/min Me_2_SO and three hearts were used for viability testing. The CT values of a representative and defined oval region in the left ventricular wall were analyzed for each heart; this field of interest was 4.29 mm^2^ in size. For each heart, the CT values of this area were calculated and plotted against the wash-in (contrast enhancement curve) and wash-out duration. Normalization was done by subtracting the CT value from the first reconstructed image (baseline), without any contrast agent present, from all the image sets in the time series. Mono-exponential fitting of the plotted CT values over time upon wash-in as well as wash-out was performed to obtain time constants for the concentration increases and decreases in the perfused substance. The dataset is provided as S1 File. Statistical significance was evaluated using the appropriate version of Student’s *t*-test, calculated using the PRISM program (GraphPad Software, San Diego, California, USA); *p* < 0.05 was considered statistically significant.

## Results

### CT values during Me_2_SO wash-in and wash-out

**Fig 1:**
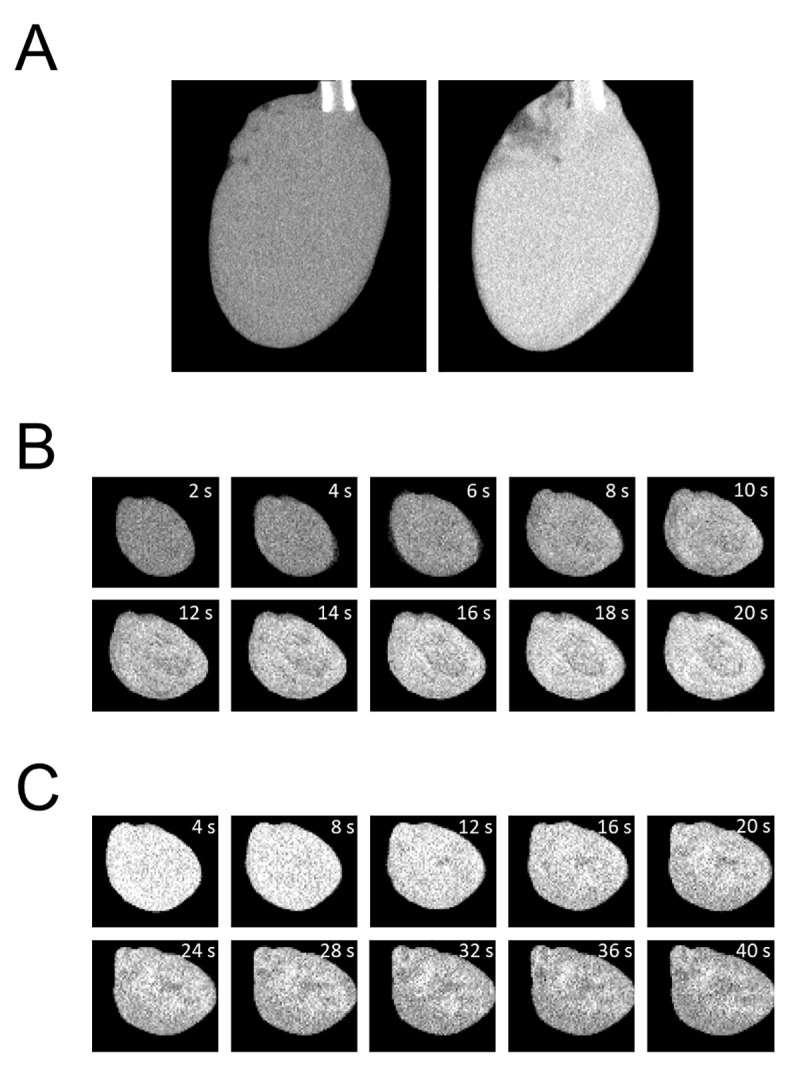
Dynamic contrast-enhanced micro-CT images of rat hearts connected to the perfusion system. Before perfusion (A, left) and 5 min after perfusion with 15% (v/v) dimethyl sulfoxide (A, right). (B) shows serial images from a representative rat heart during dimethyl sulfoxide perfusion (15% v/v) at a perfusion rate of 5 mL/min, with one reconstructed micro-CT image every 2 seconds. The dimethyl sulfoxide wash-out, at a perfusion rate of 5 mL/min, is shown in (C); serial dynamic contrast-enhanced micro-CT images, with one reconstructed image every 2 seconds (every second image is depicted).

To investigate the time course and extent of Me_2_SO distribution in tissue during perfusion, the radiographic contrast provided by Me_2_SO was taken advantage of, which is considerably higher than that of water or cardiac tissue. Fig 1A shows a DCE micro-CT image before (left) and 5 min after (right) perfusion of the heart with 15% Me_2_SO at a perfusion rate of 5 mL/min. In controlled conditions prior to Me_2_SO perfusion, the CT value in the ventricular wall was about 50 HU, a typical value for muscle tissue [8]. Five minutes after perfusion, the CT value of the heart had considerably increased to about 400 HU, which is close to the level of 15% Me_2_SO alone, suggesting complete and even distribution of Me_2_SO in all water compartments within 5 min. The experimental procedure was therefore rearranged, with the heart being perfused with 15% Me_2_SO at 5 mL/min in the CT scanner with continuous evaluation of CT images every 2 s. Fig 1B shows ten images reconstructed every 2 s from the start of perfusion. To assess the reversibility of the Me_2_SO tissue distribution, the heart was subsequently perfused with modified Tyrode’s solution free of Me_2_SO (wash-out) at 5 mL/min during continuous recording of CT images. Fig 1C shows ten images at 4-s intervals during the wash-out perfusion; every second reconstructed image is depicted. The CT values declined continuously and reached almost baseline levels after 40 s, indicating almost complete removal of Me_2_SO from the cardiac tissue.

**Fig 2:**
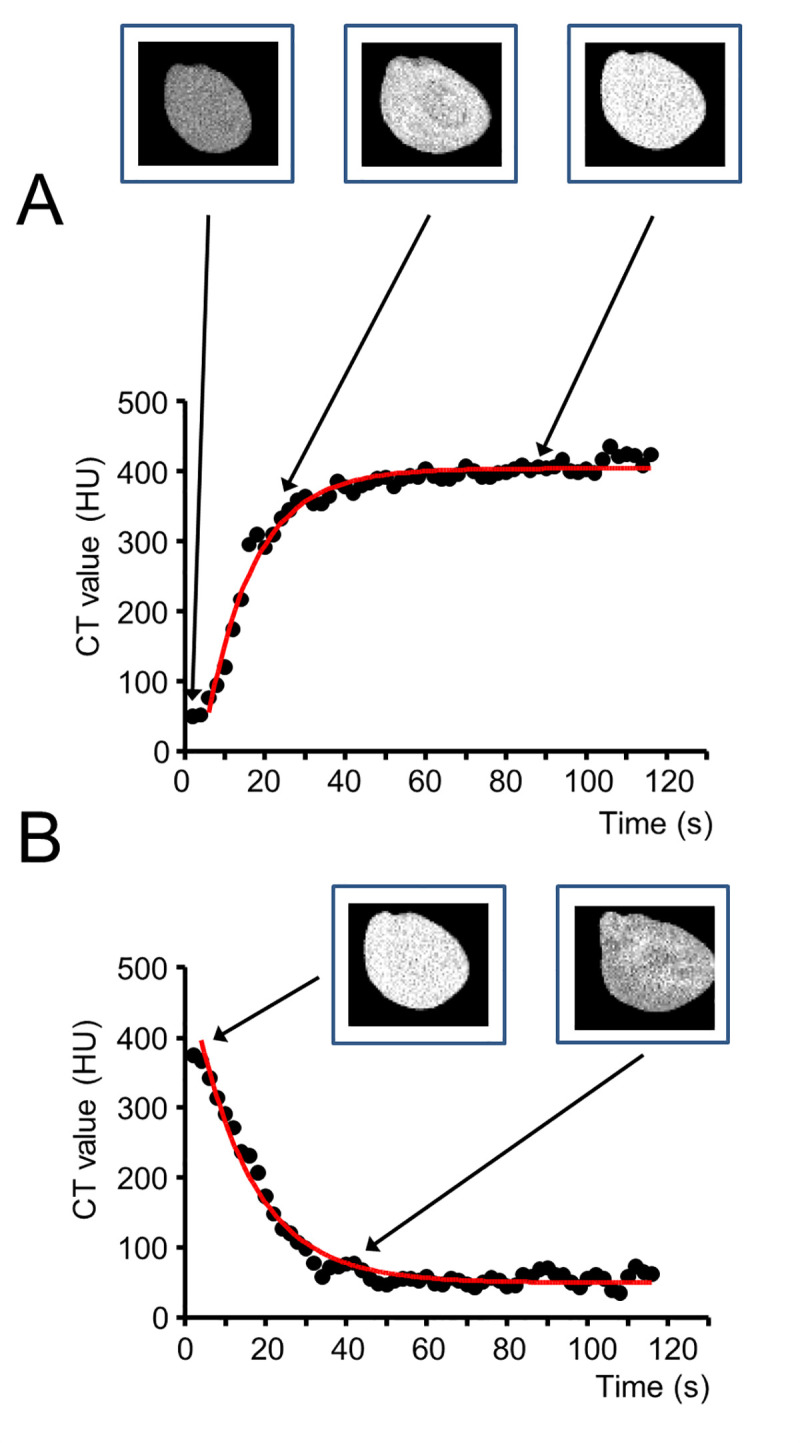
Quantitative analysis of dynamic contrast-enhanced micro-CT images of rat hearts during dimethyl sulfoxide (Me_2_SO) perfusion. (A) shows the wash-in (contrast-enhancement) curve during rat heart perfusion with 15% (v/v) Me_2_SO at a perfusion rate of 5 mL/min, measured using contrast-enhanced micro-CT. (B) depicts identical measurements during subsequent wash-out of Me_2_SO at a perfusion rate of 5 mL/min. Mono-exponential fitting lines are shown in red; the time constant for the increase in the CT value during wash-in was 11.8 s, and the decrease in CT values during wash-out was 14.7 s. Values for one representative rat heart are shown (raw data, not normalized to the initial state before Me_2_SO perfusion), linked to the associated CT images for selected time points.

Fig 2 shows a quantitative analysis of the images shown in Fig 1B and 1C. The CT value of a 4.29-mm^2^ oval region in the left ventricular wall was calculated and plotted against the elapsed time during washing in (Fig 2A) and washing out (Fig 2B) of Me_2_SO. During the wash-in, the increase in CT values followed an exponential function with a time constant (τ) of 11.8 s. Similarly, the decrease in CT values during washing out of Me_2_SO also followed an exponential function, with a slightly longer time constant of τ = 14.7 s. The calculated mono-exponential function fitted to the recorded CT values of the wash-in and wash-out data is shown in red (Fig 2A and 2B).

**Fig 3:**
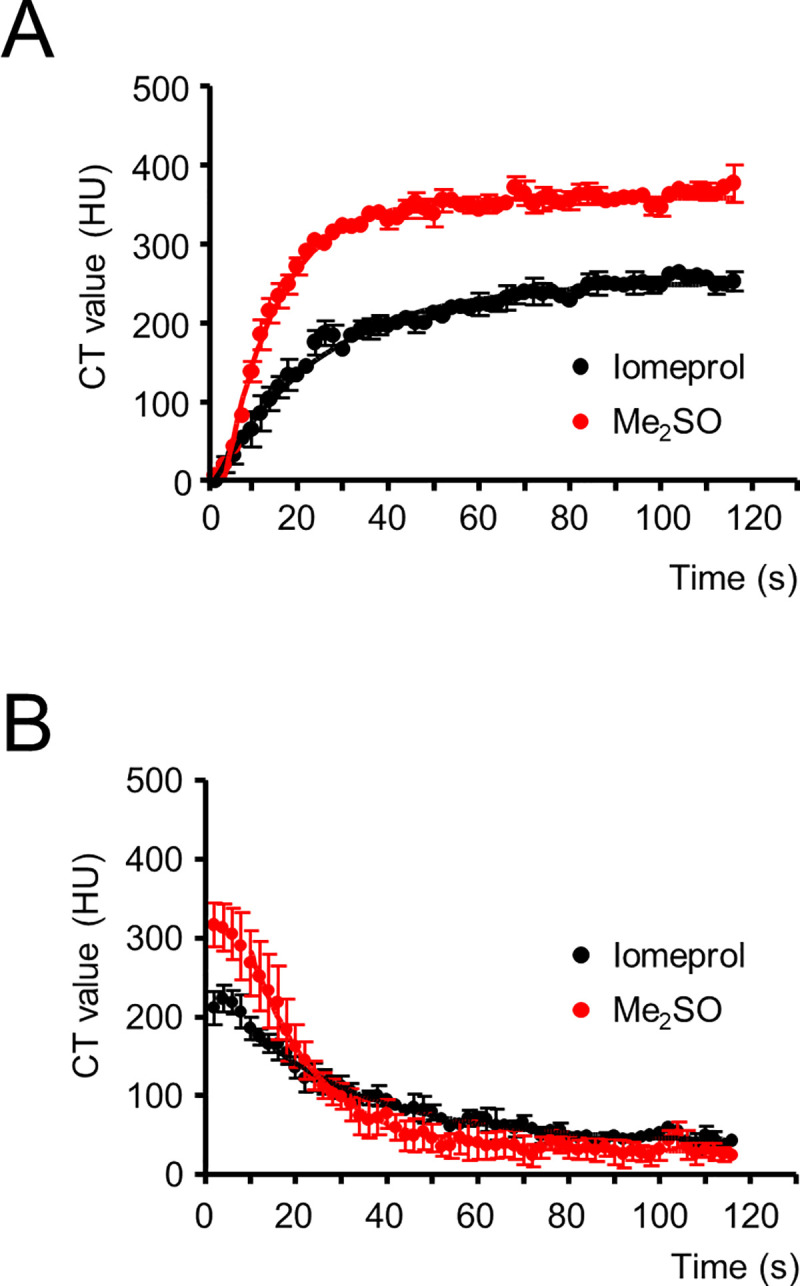
Quantitative analysis of dynamic contrast-enhanced micro-CT images of rat hearts during perfusion with dimethyl sulfoxide (Me_2_SO) and iomeprol. The wash-in (contrast-enhancement) curves of 15% (v/v) Me_2_SO (red) in comparison with 1:40 iomeprol (black; A) and the wash-out curves for both substances (B) are shown, as well as the mean values +/- SEM for dynamic contrast-enhanced micro-CT imaging data of four rat hearts (Me_2_SO) and three rat hearts (iomeprol). The iomeprol concentration was adjusted to the CT value of the 15% (v/v) Me_2_SO solution; both solutions showed a CT value of approximately 450 HU before the perfusion (individually checked for each solution before any intervention). The perfusion rates during wash-in and wash-out were 5 mL/min. Time constants after mono-exponential fitting were 11.7 ± 0.9 s for Me_2_SO wash-in and 18.1 ± 1.8 s for iomeprol wash-in (significantly different, *p* < 0.05) and 16.5 ± 3.1 s for Me_2_SO wash-out and 26.6 ± 1.5 s for iomeprol wash-out (significantly different, *p* < 0.05).

Fig 3 summarizes similar experiments. Maximum CT values (400 HU; normalized 350 HU) were reached within ≈ 36 s during Me_2_SO wash-in at a perfusion rate of 5 mL/min; further perfusion only enhanced CT values marginally (Figs 2A and 3A). Washing out of Me_2_SO at a perfusion rate of 5 mL/min took somewhat longer; the CT values decreased to almost baseline levels again within ≈ 50 s (Figs 2B and 3B). Mono-exponential fitting of the increase in CT values onto Me_2_SO wash-in yielded an average time constant of 11.7 ± 0.9 s (n = 4; Fig 3A, red); this means that tissue equilibration of Me_2_SO reached 95% after ≈ 35 s. The average time constant for the decrease in CT values during Me_2_SO wash-out was 16.5 ± 3.1 s (n = 4; Fig 3B, red), corresponding to 5% residual Me_2_SO after ≈ 49 s of wash-out.

### Me_2_SO is distributed in the extracellular and intracellular space equally and rapidly

The high CT values in the ventricular wall obtained during perfusion with Me_2_SO suggest an even distribution through the extracellular and intracellular space. To further investigate this, the CT values observed during 5 mL/min perfusion with 15% Me_2_SO solution were compared to CT values for the routinely used contrast medium iomeprol, which is quickly distributed in the extracellular space but does not enter the cells [7]. The iomeprol solutions used for perfusion were diluted to the same CT value as the 15% Me_2_SO solutions, ≈ 450 HU (solutions only). As expected, during perfusion with iomeprol solutions, the increase in CT values was substantially lower than during perfusion with the Me_2_SO solutions and averaged 253 ± 9 HU (n = 3) during the final ten seconds of perfusion compared to 367 ± 8 HU (n = 4, *p* < 0.001) using Me_2_SO solution (Fig 3A); the maximum CT value that is reached by Iomeprol is approximately 69% of the maximum Me_2_SO CT value. This indicates that iomeprol distributes into a smaller water compartment than Me_2_SO–i.e., the extracellular space, but not the intracellular space. Mono-exponential fitting of the increase in CT values after iomeprol wash-in yielded an average time constant of 18.1 ± 1.8 s (n = 3; Fig 3A, black), while the average time constant for the CT value decrease was 26.6 ± 1.5 s (n = 3; Fig 3B, black). Both time constants were significantly larger than those observed for Me_2_SO (*p < 0*.*05* for wash-in and wash-out), which is consistent with the fact that the molecular weight of iomeprol is ten times larger (777.09 g/mol) than that of Me_2_SO (78.13 g/mol).

### Me_2_SO tissue equilibration is limited by diffusion rather than perfusion

**Fig 4:**
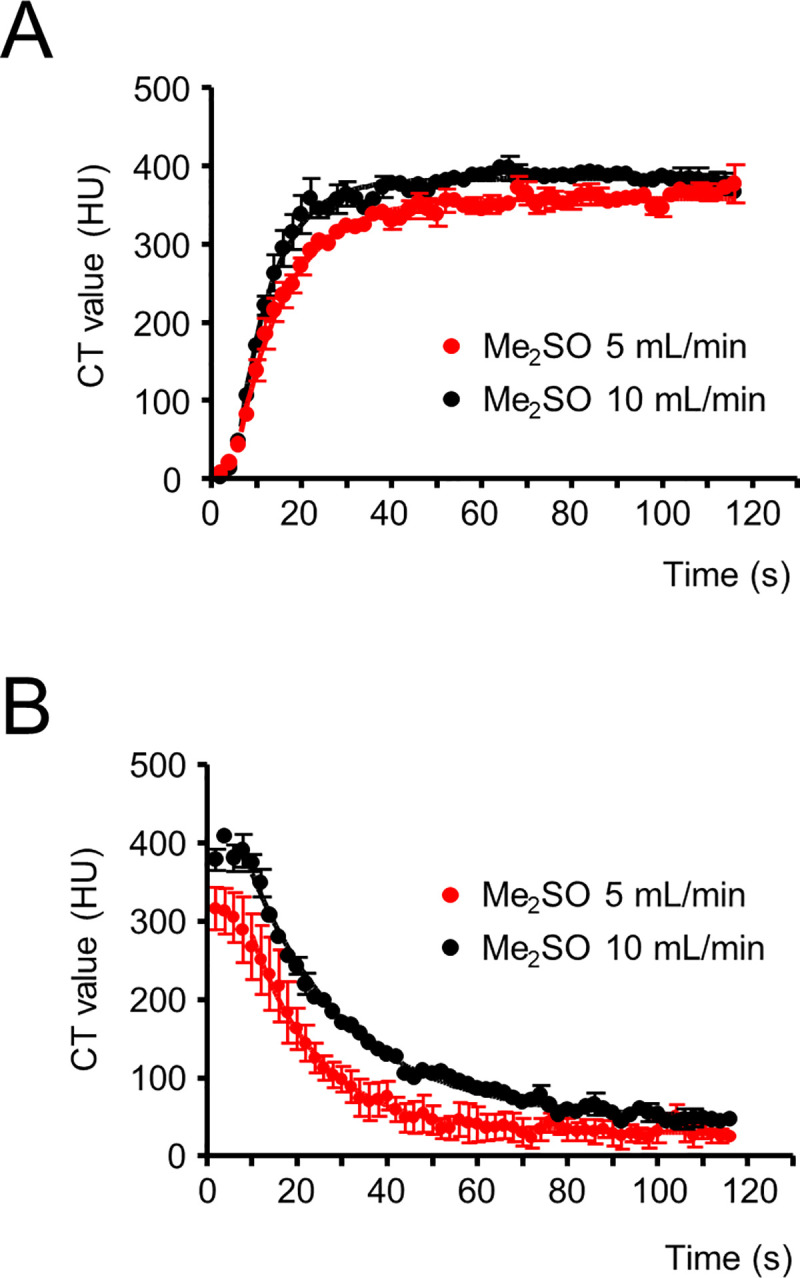
Quantitative analysis of dynamic contrast-enhanced micro-CT images of rat hearts, comparing different dimethyl sulfoxide (Me_2_SO) perfusion rates. Contrast-enhancement (wash-in) curves for 15% (v/v) Me_2_SO solution at two different perfusion rates are shown in (A)– 5 mL/min (red) and 10 mL/min (black). Wash-out curves for the two perfusion rates are given in (B). Dynamic contrast-enhanced micro-CT imaging data for four (5 mL/min) and three (10 mL/min) rat hearts are depicted, with mean values +/- SEM. The time constants obtained by mono-exponential fitting were 11.7 ± 0.9 s for 5 mL/min wash-in, 9.2 ± 0.8 s for 10 mL/min wash-in (no significant differences, *p* = 0.11) and 16.5 ± 3.1 s for 5 mL/min wash-out and 22.0 ± 2.9 s for 10 mL/min wash-out (no significant differences, *p* = 0.27).

To find out whether Me_2_SO equilibration in the whole organ is limited by perfusion rather than by diffusion, Me_2_SO perfusion experiments were repeated with the same Me_2_SO concentration, but at an increased perfusion rate of 10 mL/min. The CT values obtained during the two perfusion rates are depicted in Fig 4A (wash-in) and 4B (wash-out). The maximum CT values recorded during wash-in and the minimum CT values recorded during wash-out were similar for the two perfusion rates. Mono-exponential fitting of the increase in CT values after wash-in yielded an average time constant of 9.2 ± 0.8 s (n = 3; Fig 4A, black) for the higher perfusion rate (10 mL/min), which was slightly, but not significantly, shorter in comparison with the average time constant of 11.7 ± 0.9 s (n = 4; Fig 4A, red; *P* = 0.11) for the lower perfusion rate (5 mL/min). The average time constant for the decrease in CT values during wash-out was 22.0 ± 2.9 s (n = 3; Fig 4B, black) for the higher perfusion rate (10 mL/min) and was slightly, but not significantly, longer in comparison with the lower perfusion rate (16.5 ± 3.1 s; n = 4; *P* = 0.27; Fig 4B, red). These results indicate that tissue equilibration of Me_2_SO during perfusion at a rate of 5 mL/min is not limited by perfusion, but rather by diffusion of Me_2_SO.

## Discussion

Whole-organ cryopreservation would revolutionize the options available for organ donation and treatment of transplant recipients, as it would gain time to improve donor–recipient matching and therefore reduce the numbers of transplant rejections, as well as the intensity of the immunosuppression required [9]. Despite partial successes have been achieved with the kidney [10], heart [9], uterus [11–14], and ovary [15, 16] in animal models, using perfusion-based equilibration protocols for CPAs as previously reviewed [17], the current freezing techniques do not ensure the survival and function of organs and they are far from being ready for routine clinical applications [18]. Most of the perfusion-based protocols for organ cryopreservation that have been published share the common feature that the CPA equilibration durations used range from approximately 25 minutes up to a couple of hours. Passive equilibration using diffusion alone would increase the recommended equilibration duration even further: Corral et al. [19] postulated that passive whole-organ equilibration of a rabbit kidney with Me_2_SO would take 7–9 days. None of the above studies carried out real-time monitoring of the equilibration process; they either estimated CPA tissue concentrations based on indirect reference values ([10]), or evaluated the CPA concentration after assumed equilibration [15, 19]–leaving open the question of whether the Me_2_SO distribution actually reaches an adequate level much earlier. The present study shows that all cells and extracellular spaces in rat heart tissue are 95% equilibrated after ≈ 35 s perfusion with 15% (v/v) Me_2_SO; thus, Me_2_SO tissue distribution completes much more rapidly upon organ perfusion than previously expected. This implies that the equilibration duration with Me_2_SO usually assumed is too long, at least for perfusion-based equilibrations. The possibility needs to be taken into account that long equilibration periods may contribute to tissue damage, due to toxicity during freeze–thawing procedures [2].

In general, it would be valuable to observe CPA tissue concentrations in real-time to monitor equilibration status, as this would make it possible to adjust both equilibration duration and also the initial CPA concentrations in the equilibration solution. In the case of Me_2_SO, such real-time observation is possible using CT, due to the high electron density of the sulfur atom included [19]. Herein, we could confirm that real-time CT-imaging is a useful tool to assess Me_2_SO tissue distribution. Besides real-time observation, another advantage of using dynamic contrast-enhanced micro-CT to evaluate Me_2_SO tissue distribution is that the technique is rather non-invasive, i.e. there is no necessity for biopsies or other invasive procedures. Hence, organ architecture remains untouched. On the other hand, if CT-based evaluation would be routinely used during organ conservation procedures, it would considerably increase the time from organ harvesting until perfusion with the cryoprotectant. Therefore, we suggest CT-based analysis of Me_2_SO tissue distribution as a useful tool to optimize and validate cryopreservation protocols, rather than a routinely used procedure. In this respect, one should keep in mind that CT evaluation requires a certain intensity of radiation, which, depending on the tissue or organ, may be harmful.

Tissue cryopreservation techniques are more advanced than whole-organ preservation and in some cases are already in use in routine practice–for example, the freezing of ovarian tissue for the purpose of preserving fertility [20–23]. The methods currently used for cryopreservation of ovarian tissue were empirically adapted from the protocols for freezing gametes and embryos [24]; however, it is difficult to ensure full penetration of a CPA via diffusion in tissue pieces using parameters adapted from single-cell protocols. Tissue damage after freeze–thawing cycles still occurs with the current protocols [25]. Techniques for preserving ovarian tissue therefore still need to be improved. Long equilibration periods with CPAs may contribute to toxicity-induced tissue damage [2]. Particularly in the case of Me_2_SO, which is one of the most frequently used CPAs for fertility preservation [26], it might be possible to apply a shorter equilibration duration given the fast penetration of Me_2_SO observed in the present study.

Corral et al. recently tried to optimize freezing protocols for human ovarian tissue in two studies, using diffusion-based equilibration protocols in a bovine model [26, 27]. Me_2_SO concentrations inside the frozen tissue were analyzed using X-ray CT with frozen specimens. Unfortunately, this was done without monitoring the penetration of Me_2_SO during the equilibration phases, which ranged from 70 to 190 minutes in [27], or lasted approximately 18 minutes (CPA incubation prior to ice seeding) in [26]. In addition, toxicity was not evaluated in [26]; in [27], two groups with shorter equilibration duration (70 and 85 minutes) were excluded from further histological analysis because the final Me_2_SO concentration was regarded as too low. No information was consequently available on whether the final equilibration status of the tissue would have been sufficient to protect it from freezing damage. Shorter Me_2_SO incubation duration during ovarian tissue preservation could potentially be sufficient and advantageous. In addition, a perfusion-based CPA equilibration process might have been more beneficial in relation to equilibration time and toxicity.

Perfusion based CPA procedures which use supply routes that already exist, such as the vascular system, should replace the passive and time-consuming process of diffusion-based CPA equilibration, where feasible; particularly in case of larger tissue pieces or organs. In the case of the ovaries, this has already been done by Campbell et al. [15], who evaluated the restoration of full organ function after whole-ovary freeze–thawing following autotransplantation in a sheep model. Equilibration was carried out via perfusion with 1.5 M Me_2_SO prior to freezing; however, 10 minutes of perfusion was inferior to 60 minutes [15]. This much longer equilibration period than the ≈ 35 s required for 95% tissue equilibration in the present study, with an only slightly lower Me_2_SO concentration, might be due to the fact that the perfusion rate used by Campbell et al. was 10 times lower; Campbell et al. used 0.5 mL/min, while a perfusion rate of at least 5 mL/min was used in the present study. It is conceivable that a perfusion rate higher than 0.5 mL/min could shorten the perfusion time required to achieve full equilibration, as the present perfusion rates were able to equilibrate rat heart tissue to 95% within ≈ 35 s. In addition, rat hearts are smaller than sheep ovaries and this can presumably also influence the equilibration time required. The perfusion temperatures used also differed. Campbell et al. perfused on ice, whereas perfusion at 22°C was used in the present study. Higher temperatures lead to faster Me_2_SO diffusion rates– 1.4–3.7-fold faster, depending on temperature difference and tissue type [28, 29]. Differences in the duration of perfusion-based equilibration may also depend on the organ type, since another factor in Me_2_SO penetration is the tissue type [28–30]. The heart and ovary have completely different tissue-type characteristics; the heart mainly consists of myocardium, whereas the main component of the ovaries is connective tissue, in addition to follicles [31]. The heart and ovary also differ in relation to the microarchitecture of the vascular system–resulting in a high rate of physiological blood flow in the heart, which is ≈ 2.4–3-fold higher per gram of tissue than in the ovary [32]. Consequently, perfusion-based CPA equilibration of an organ that is designed for high blood flow will theoretically take less time to reach individual cells. In addition to these differences in average blood flow in the whole organ, the ovary has further intraorgan differences in blood supply. The cortex region, which contains the majority of resting follicles, simultaneously represents the most poorly vascularized zone in the whole ovary [33]. It can, therefore, be assumed that this cortical area is at a disadvantage during perfusion-based CPA equilibration, resulting in a need for longer perfusion duration in order to fully equilibrate and protect resting follicles. This would additionally explain why 60 minutes of Me_2_SO perfusion time was superior to 10 minutes in relation to the survival of primordial follicles in the study by Campbell et al. [15].

Results from the two studies can therefore not be adapted on a one-to-one basis for a different organ. In future evaluations of perfusion-based CPA equilibrations, it might be beneficial to focus on different perfusion temperatures, perfusion rates and perfusion durations, while monitoring the penetration of CPAs in real-time during the equilibration process. However, some aspects need to be considered. Even organs with a high relative blood flow might contain areas with lower levels of perfusion and Me_2_SO saturation in these areas might take more time. This might be particularly relevant in organs with substantial regional differences in blood flow, such as the kidney. Future studies using spiral CT scans and larger animal models could address this question.

The fact that Me_2_SO perfusion leads to a 1.44-fold higher maximum CT value than iomeprol (360 HU vs. 250 HU), by using solutions with adjusted CT values, indicates that Me_2_SO leaves the extracellular space and enters the cells. If 450 HU (Me_2_SO and iomeprol, solutions only) is set as 100%, then 350 HU during the steady-state of Me_2_SO corresponds to ≈ 78%; this precisely matches the water content of rat heart tissue, which is 77.9 ± 0.2% [34]. During the steady-state in the case of iomeprol, 250 HU corresponds to 51%. Since intracellular fluid represents approximately 40% of the total weight of the organ [35], 30–40% was expected rather than approximately 51% for iomeprol perfusion. A potential explanation for the higher value might be the development of edema during the perfusion process. The development of edema in Langendorff-perfused hearts usually occurs due to vasodilation, which increases the capillary perfusion pressure [36]. In addition, oncotic pressure decreases due to a lack of large plasma proteins in the iomeprol perfusion solution–lowering the oncotic pressure and thus leading to increased fluid secretion into the interstitial space.

CT values reached a minimum of about 50 HU during wash-out processes and maintain that level up to the time point of 120 seconds (Fig 3B). Small residues of Me_2_SO and iomeprol therefore appear to remain in the tissue after wash-out with modified Tyrode’s solution. In the case of Me_2_SO, this was also previously suspected by Fuller et al. [37] based on a similar observation. With Me_2_SO, this residual CT contrast is most probably a consequence of the Me_2_SO binding to globular proteins [38, 39]. In the case of iomeprol, the remaining CT contrast might be due to tissue swelling during perfusion, as suggested above.

The performed cell viability assay in the present study indicated that cardiomyocytes were not significantly affected by Me_2_SO -wash-in and wash-out under our experimental conditions. This is supported by results of Fahy and Karow [40] who reported that isolated rat hearts maintained 30% of their initial contractile strength after 20 minutes perfusion with ‘balanced salt solution’ containing 15% Me_2_SO and subsequent wash-out.

Lovelock and Bishop [3] reported that 30 seconds of equilibration time in 15% (w/w) Me_2_SO was sufficient to completely protect red blood cells during subsequent freezing. Interestingly, this passive equilibration via diffusion of cells in suspension was not that much faster than the equilibration of perfused rat heart tissue in the present study. This suggests the hypothesis that single cells in a whole organ can be reached very quickly via perfusion and that the equilibration process is then limited by subsequent passive diffusion into single cells. This hypothesis is supported by the fact that the curved shapes of Me_2_SO tissue infiltration during wash-in in the present study are similar to those described by Newton et al. [29], who monitored Me_2_SO tissue penetration during diffusion–implying that we also generally see a diffusion process during wash-in. In perfusion-driven support, the Me_2_SO solution is simultaneously transported to multiple points for subsequent diffusion inside the cells, as the final equilibration step–saving all the time that would be needed to reach all of the cells through passive diffusion alone, particularly in deeper parts of the tissue. It would generally be advisable to focus on perfusion-based–or at least perfusion-supported–equilibration procedures.

## Conclusion

The entire equilibration process that takes place in routine Me_2_SO-based cryopreservation protocols need to be called into question since Me_2_SO penetration appears to be much more rapid than has previously been assumed. Future studies both on reducing incubation duration and on decreasing CPA concentrations are necessary. Switching to perfusion-based equilibration procedures may be beneficial, if feasible, taking into account differences in the architecture of various organs and tissues. In the case of Me_2_SO, CT imaging is an advantageous and non-invasive technique for monitoring the equilibration process in real-time. The information obtained could be directly linked to tissue survival after freeze–thawing, and also to residual functionality, and could also serve as a basis for improving tissue-preservation and organ-preservation protocols.

## Supporting information

S1 File Dataset. Computed tomography (CT) values over time during wash-in and wash-out for each perfusion are provided. Normalized CT values (CT value from the first reconstructed image (baseline) subtracted) and the Mono-exponential fitting of the plotted CT values over time upon wash-in as well as wash-out are given as well.
(XLSX)
